# A simple and reliable PCR-based method to differentiate between XX and XY sex genotypes in *Cannabis sativa*

**DOI:** 10.1007/s00425-025-04804-z

**Published:** 2025-08-22

**Authors:** Ainhoa Riera-Begue, Matteo Toscani, Afsheen Malik, Caroline A. Dowling, Susanne Schilling, Rainer Melzer

**Affiliations:** 1 UCD School of Biology and Environmental Sciences and UCD Earth Institute, University College Dublin, Dublin, Ireland; 2 Present Address: Lancaster Environment Centre, Lancaster University, Lancaster, LA1 4YQ UK

**Keywords:** Cannabis sativa, CAPS, Dioecious, Hemp, Sex chromosome, Sex determination, Sex marker, TaqMan

## Abstract

*Cannabis sativa* is a mainly dioecious plant, which means that female and male flowers develop on separate individuals, which is controlled by an XY sex determination system: females have two X chromosomes and male plants carry an X and a Y chromosome. *C. sativa* is a crop that has a wide variety of applications, some of which depend on the sex of the plant. Females are, for example, used for the cannabinoid production, as cannabinoids are produced in the female inflorescence. However, while adult *C. sativa* individuals present high sexual dimorphism, it is not possible to phenotypically distinguish male and female plants before flowering. Here we present the identification of a highly conserved sex marker, *CsPDS5*, and the development of a robust, reliable and affordable PCR-based method to determine the sex genotype. In contrast to other sex genotyping methods, our approach relies on a gene polymorphic between the X and Y chromosomes and therefore requires only a single PCR with one pair of primers. The method was tested in 14 hemp-type cultivars and 6 crosses, with different tissues and developmental stages, on more than 500 samples, with 100% accuracy. Our assay allows early sex identification and hemp selection, which is useful for both research and industrial purposes. Finally, the pipeline presented here to identify genes polymorphic between the X and Y chromosomes can serve to discover new sex markers, not only in *C. sativa* but also in other dioecious plants and other organisms with 2 sexes.

**Supplementary Information:**

The online version contains supplementary material available at 10.1007/s00425-025-04804-z.

## Introduction

Most flowering plants are bisexual, meaning flowers have both male and female reproductive organs. However, about 6% of the angiosperms develop male and female flowers on separate individuals, a phenomenon called dioecy (Renner 2014). *Cannabis sativa* is mainly a dioecious plant, although there are some monoecious cultivars with female and male flowers on the same individual (Moliterni et al. 2004). *C. sativa* is diploid with a pair of sex chromosomes; hence, sex is primarily determined by an XY chromosome system, female and monoecious plants are XX and males are XY (Moliterni et al. 2004).

*C. sativa* is a multipurpose crop with a wide spectrum of uses, ranging from textiles and building materials that derive from the stalks, biofuel and oil from the seeds, to medicinal and recreational purposes thanks to the cannabinoids present in the female flower’s trichomes (Schilling et al. 2020). Depending on the intended use, female plants are preferred, e.g. for pharmacological applications, or male plants, e.g. for industrial applications due to their high-quality fibre content (Salentijn et al. 2019). Adult *C. sativa* individuals show a high degree of dimorphism (Petit et al. 2020), which makes it easy to differentiate between male and female plants phenotypically. However, at early stages of development before the onset of flowering, this sexual dimorphism is not present (Shi et al. 2024). Thus, for research and industrial purposes, there is a need for reliable methods to sex genotype the plants before flowering.

In recent years, different methods of sex genotyping have been developed. In most cases, MADC (male-associated DNA Cannabis) sequences have been used as markers with different strategies (Mandolino et al. 1999; Techen et al. 2010; Toth et al. 2020; Torres et al. 2022), some of them with great accuracy. However, it must be taken into account that MADC sequences are only associated with male individuals, presumably because they are located on the Y chromosome. Used as PCR markers, they therefore only result in amplification of male (Y chromosomal) DNA, and an autosomal control is always needed for the female or monoecious genotypes lacking a Y chromosome. Furthermore, MADC6 and some other markers are located in retrotransposons, so they may not be present in all cultivars, potentially reducing the marker’s reliability (Toth et al. 2020). Prentout et al. 2025 tried to solve this problem by identifying Y-linked genes. However, also in this assay, autosomal controls were required. Gilchrist et al. (2023) identified SNPs between the X and the Y chromosome that can be leveraged in high resolution melting analyses to distinguish male and female individuals. This is a promising approach but requires specialised instruments and it is not clear how conserved the identified SNPs are across different *C. sativa* cultivars.

Hence, there is still a need to identify a universal genetic marker for sexing *C. sativa* seedlings. Here we present an affordable, reliable and robust PCR-based CAPS (Cleaved Amplified Polymorphic Sequence) method to differentiate between XX and XY genotypes in *C. sativa* across cultivars, as well as a pipeline that can be used to identify markers in other dioecious species.

## Results

At the time of this study, no male *C. sativa* genome assembly was publicly available. The only available genome from a male individual, ‘JL_father’ (GCA_013030025.1), was assembled at the scaffold level, making it impossible to easily identify Y chromosomal sequences.

To obtain sequences which likely originated from the Y chromosome, we previously described a computational pipeline (Shi et al. 2025). The pipeline uses RNA-seq data and hence does not rely on genome assemblies containing a Y chromosome. Briefly, we used RNA-seq samples from male and female *C. sativa* plants from the same developmental stage and used the following criteria to identify putative Y chromosomal transcripts.

Transcripts were required to

1. not map to the female CBDRx (GCA_900626175.2) genome.
2. have a sequence signature (k-mer of 16 nucleotides) shared by all male samples but different from all female samples.
3. be male biased in their expression.

The pipeline resulted in the initial identification of 379 transcripts putatively originating from the Y chromosome (Shi et al. 2025).

To build on the Shi et al. 2025 method and further reduce the inclusion of false positives, a strict TPM expression level filter (average TPM > 10 in male samples, and TPM = 0 in all female samples) was applied. This allowed us to identify 25 transcripts that originated from the Y chromosome with relatively high certainty (Supplementary Data S1). From those transcripts, the genomic sequence was retrieved through a BLAST search of the ‘JL_father’ assembly. Even though this genome is not assembled at the chromosome level, blasting the mRNA sequences of the 25 putatively Y chromosomal transcripts allowed us to identify scaffolds that are most likely part of the Y chromosome. The genomic sequences of the 25 genes were then aligned to putatively corresponding sections on the X chromosome identified through a BLAST search of the CBDRx genome assembly (GCA_900626175.2). The genes with the highest sequence divergence between X and Y that were deemed the most suitable for a PCR assay to distinguish XX and XY genotypes were FE.chrY.t5, FE.chrY.t9, FE.chrY.t122, FE.chrY.t25, FE.chrY.t42 and FE.chrY.t47 (Supplementary Data S2, transcript naming after Shi et al. 2025). Of those, we focussed on FE.chrY.t9.

FE.chrY.t9 is homologous to the *PRECOCIOUS DISSOCIATION OF SISTERS 5* (*PDS5*) gene from *Arabidopsis thaliana* and was therefore renamed *CsPDS5*. This gene is positioned at 80.6 Mbp in chromosome X in what we believe is an ancient stratum (Toscani et al. 2025)*.* PDS5 proteins play a crucial role in regulating genome architecture by influencing the 3D chromatin organisation; the *A. thaliana PDS5* genes encode cohesin cofactors, the depletion of which compromises development, fertility and homologous recombination (HR) during DNA repair mechanisms, and it also causes subtle meiotic alterations (Pradillo et al. 2015).

**Fig. 1:**
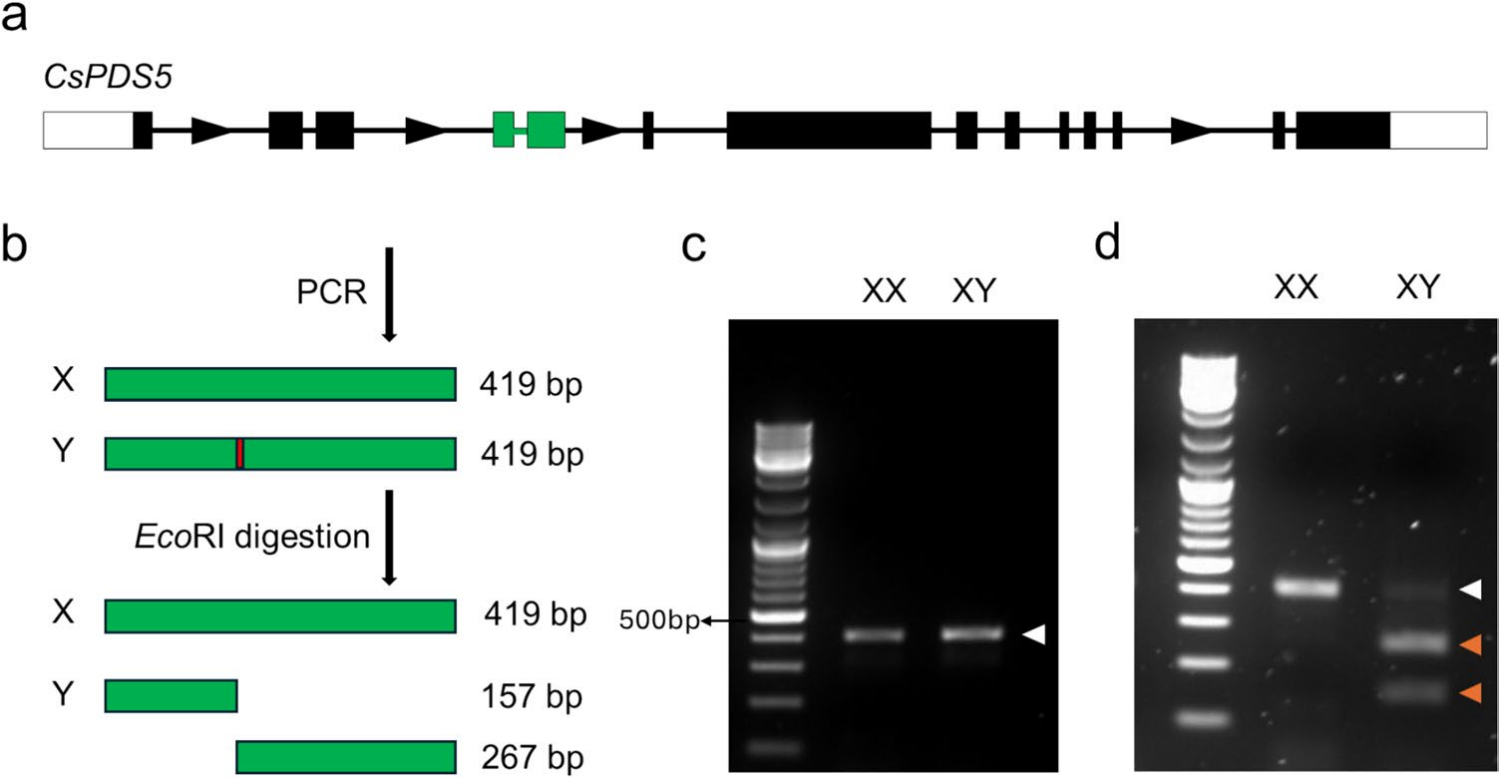
Overview over *CsPDS5-CAPS* for the *C. sativa* sex genotyping. A schematic representation of *CsPDS5* with exons and introns depicted as boxes and lines, respectively (**a**). The direction of transcription is indicated by arrows. The principle of the CAPS PCR-based genotyping showing amplicons in green and the EcoRI digestion site in red (**b**). Agarose gel electrophoresis of a female (XX) and male (XY) sample pre- (**c**) and post- (**d**) digestion. White and orange arrowheads indicate undigested (419 bp) and digested (157 and 262 bp) amplicons, respectively. Size marker: GeneRuler DNA Ladder Mix (Fermentas)

Although both the X and Y chromosome versions of *CsPDS5* are diverged, there is a highly conserved region, which allowed the design of a common pair of primers that amplified a 419 bp region from the X as well as from the Y chromosome. Importantly, the Y version contains an EcoRI restriction enzyme recognition inside the amplicon, which is not present in the X allele. Thus, a CAPS assay in which the PCR products are subjected to digestion with EcoRI results in a 157 bp and a 262 bp band for Y chromosome amplicons, while X chromosome amplicons remain at their original 419 bp length (Fig. 1). Hence, visualisation via an agarose gel results in only one band at 419 bp for female and monoecious (XX) samples, while the male (XY) samples display three bands, one corresponding to the amplicon derived from the X chromosome at 419 bp and two smaller bands at 157 bp and 262 bp, corresponding to the digested amplicon derived from the Y chromosome (Fig. 1). We term this assay *CsPDS5-CAPS* hereafter.

The assay was initially tested using the dioecious cultivar ‘FINOLA’. Leaf samples collected at the flowering stage, when sexual dimorphism was fully apparent, were used to verify that the sex as determined by *CsPDS5-CAPS* agreed with the phenotypic sex. Subsequently, ‘FINOLA’ samples at different developmental stages (from germination to flowering) and from different tissues (cotyledon and leaf) were tested, and the sex as determined by *CsPDS5-CAPS* was compared to the phenotypic sex. In total, 312 ‘FINOLA’ samples were tested, and the *CsPDS5-CAPS* genotyped sex matched the phenotypic sex in all cases.

**Fig. 2:**
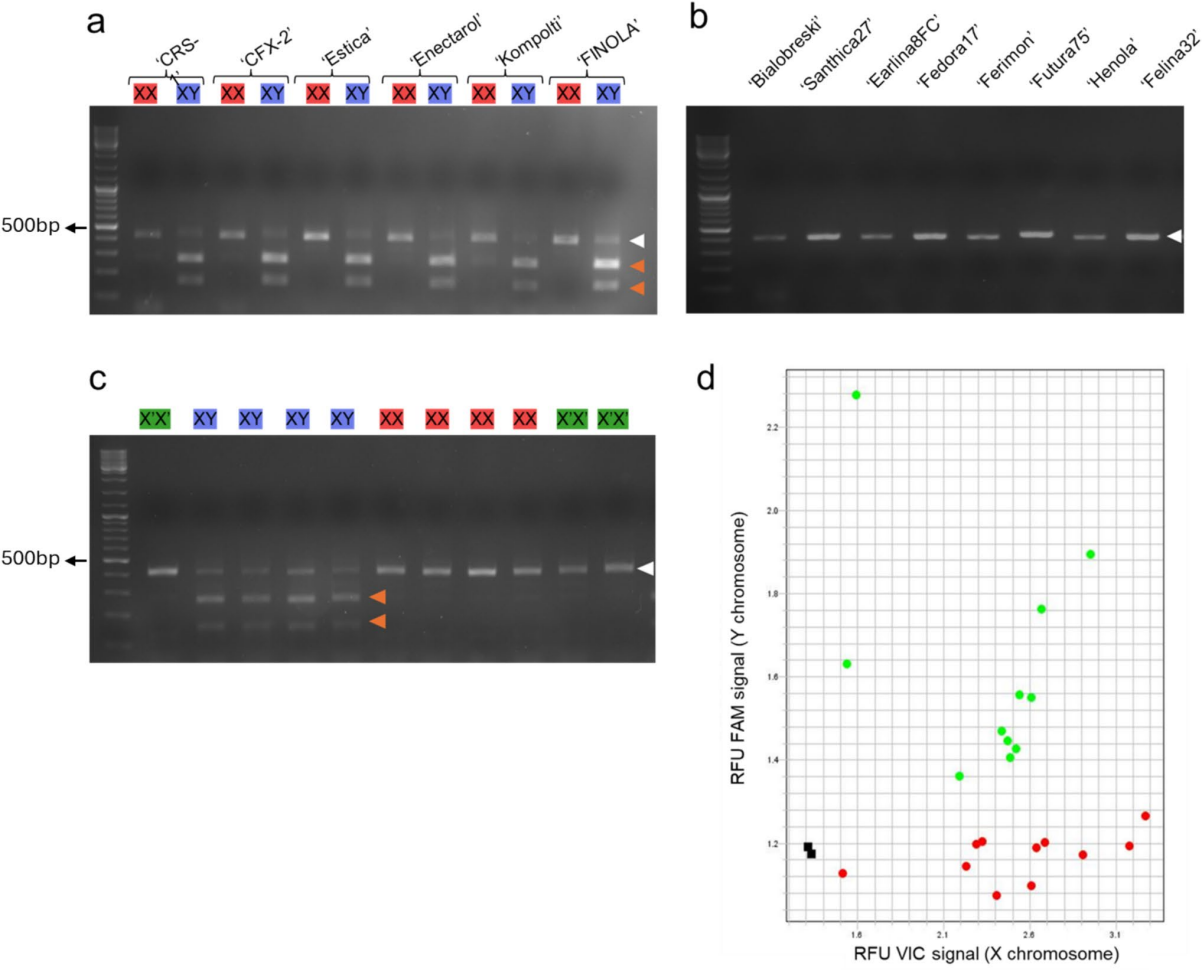
Genotyping results for different cultivars of *C. sativa*. *CsPDS5-CAPS* genotyping for female (XX) and male (XY) samples from six different dioecious cultivars (**a**), eight different monoecious cultivars (**b**) and 11 representative F2 individuals of a ‘FINOLA’ x ‘Felina 32’ cross (**c**). Cultivar names and phenotypic sex are depicted above the gel image (X’X’ = Monoecious (green), XX = Female (red) and XY = male (blue)). Gel images depict PCR fragments after EcoRI digestion. White and orange arrowheads indicate undigested (419 bp) and digested (157 and 262 bp) amplicons, respectively. Size marker: GeneRuler DNA Ladder Mix (Fermentas). Panel (d) shows the allelic discrimination plot of the 22 'FINOLA' samples analysed using the TaqMan SNP genotyping assay targeting the *CsPDS5* polymorphism. The plot displays relative fluorescence units (RFU), with the X-axis representing the VIC signal (X allele) and the Y-axis representing the FAM signal (Y allele). Red dots correspond to XX samples, green dots to XY samples and black squares represent NTCs (no template controls)

Subsequently, we assessed the performance of *CsPDS5-CAPS* for 14 different hemp-type cultivars (Figure 2, Table 1), six dioecious (‘FINOLA’, ‘Kompolti’, ‘CSR-1’, ‘CFX-2’, ‘Estica’ and ‘Enectarol’) and eight monoecious (‘Felina 32’, ‘Bialobrzeski’, ‘Santhica 27’, ‘Earlina 8 FC’, ‘Fedora 17’, ‘Ferimon’, ‘Futura 75’ and ‘Henola’). For all dioecious cultivars, our genotyping distinguished male and female individuals (Fig. 2a). Monoecious cultivars are typically reported to carry two X chromosomes (Faux et al. 2014) and consequently appeared genotypically female in our assay (Fig. 2b).

To test the suitability of *CsPDS5-CAPS* for breeding projects, we further tested the assay on progeny plants resulting from crosses between different hemp-type cultivars. For this purpose, 137 F2 individuals from a previously established ‘FINOLA’ × ‘Felina 32’ cross (Dowling et al. 2024) were tested. As ‘FINOLA’ is dioecious and ‘Felina 32’ is monoecious, the F2 population comprises male, female and monoecious individuals (Dowling et al 2024). In all 137 cases tested, our genotyping results were in agreement with the phenotypic sex (Fig. 2c). We next tested F1 individuals from five additional crosses (‘FINOLA’ × ‘Earlina 8 FC’, ‘FINOLA’ × ‘Ferimon’, ‘FINOLA’ × ‘Santhica 27’, ‘FINOLA’ × ‘Fedora 17’ and ‘Felina 32’ × ‘Estica’). Also in this case, the *CsPDS5-CAPS* results were in perfect agreement with the phenotypic sex.

In total, over 500 different samples were used to test our genotyping assay (Table 1). In all cases, the genetic and phenotypic sex matched, showing that the *CsPDS5-CAPS* is a highly reliable assay and produces robust results independently of the cultivar, crossing, developmental stage or tissue.

To improve the *CsPDS5-CAPS* assay, we adapted it into a high-throughput TaqMan assay. The method is based on a pair of primers that amplify a ≈200 bp fragment of the *CsPDS5* gene region, in both the X and Y versions, which contains the EcoRI restriction site. A male-specific probe was designed to bind at the restriction site present in the Y chromosome, while a female-specific probe binds to the corresponding sequence in the X chromosome. Each probe is labelled with a different reporter dye: the Fem probe with VIC and the Male probe with FAM. Hence, in this TaqMan PCR setup, XX samples produce only a VIC signal, while XY samples yield both VIC and FAM signals. We validated this assay with 22 ‘FINOLA’ samples (11 male and 11 female) previously genotyped using *CsPDS5-CAPS*, and the results were consistent between both approaches, as shown in the allelic discrimination plot (Fig. 2d).

**Table 1:** Summary table showing number of individuals tested per cultivar/crossing categorised by sex

Cultivar/crossing	Samples*
FINOLA	160 females 152 males
Kompolti	1 female 1 male
CRS-1	3 females 3 males
CFX-2	1 female 1 male
Estica	5 female 5 male
Enectarol	1 female 1 male
Felina 32	2 monoecious
Bialobreski	2 monoecious
Santhica 27	2 monoecious
Earlina 8 FC	2 monoecious
Fedora 17	2 monoecious
Ferimon	2 monoecious
Futura	2 monoecious
Henola	2 monoecious
Felina 32 × FINOLA	58 females 28 males 53 monoecious
FINOLA × Earlina 8FC	3 females 8 males
FINOLA × Ferimon	4 males
FINOLA × Santhica 27	2 males
FINOLA × Fedora 17	2 females 3 males
Felina 32 × Estica	6 females 5 monoecious

*All samples in the table were tested at flowering stage, except for 24 at cotyledon stage (14 females and 10 males) and 280 at 2nd/3rd leaf stage (142 females and 138 males) of the 'FINOLA' cultivar

**Fig. 3:**
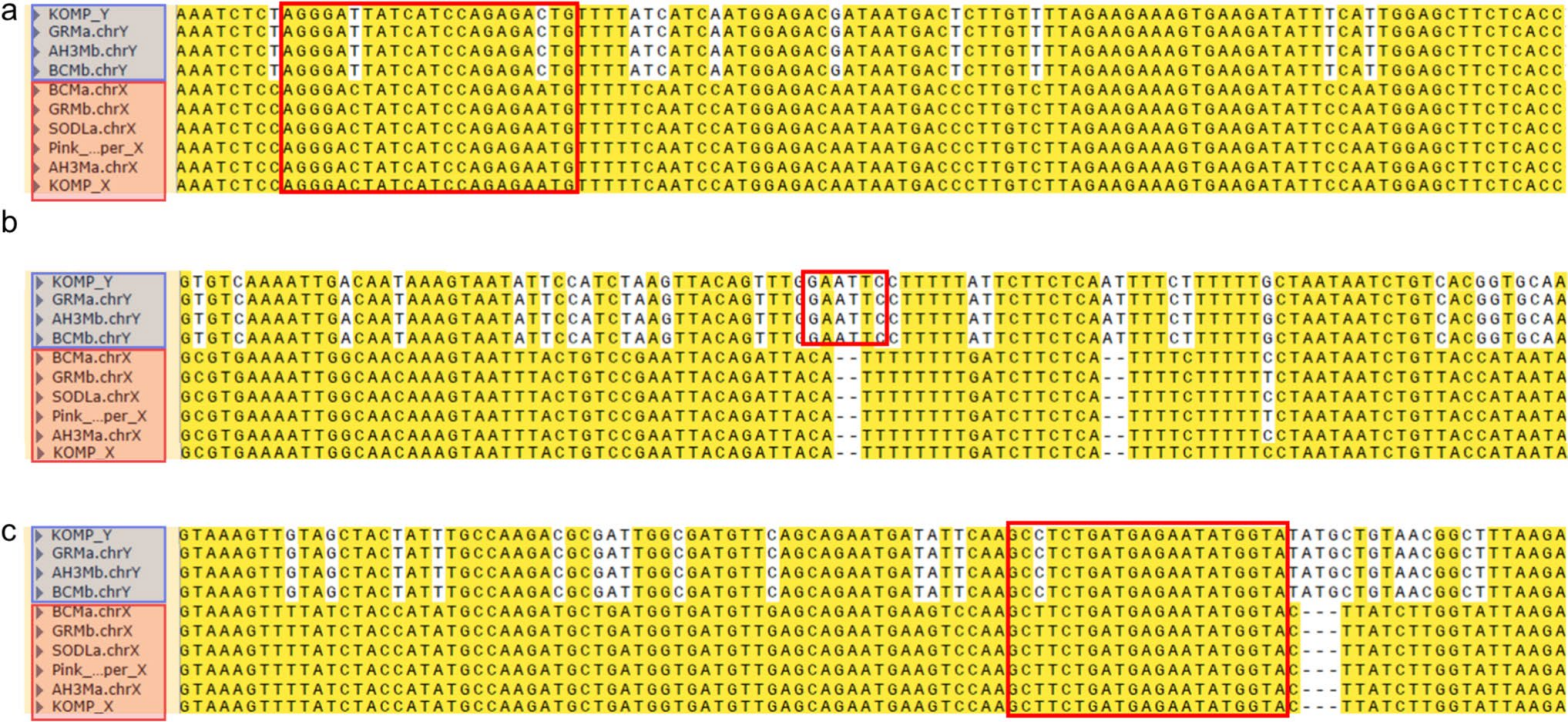
*CsPDS5* sequence from different cultivars of *C. sativa*. Alignment of three non-consecutives regions of *CsPDS5.* The red frame denotes the forward primer binding site (**a**), the EcoRI restriction site (**b**) and the reverse primer binding site (**c**). ChrX and chrY sequences from *C. sativa* cultivars ‘GoldenRedwood’ (GRM), ‘Ace High 3–2’ (AH3M), ‘BooneCounty’ (BCM), ‘SourDiesel’ (SODL) and ‘Kompolti’ (KOMP) cultivars, along with the X chromosome version of ‘Pink Pepper’ as reference genome cultivar. Bases conserved in the majority of sequences have a yellow background. SNPs in the primer binding sites were incorporated as degenerate bases in the primer sequences (Table 2)

We next analysed whether the *CsPDS5* polymorphism utilised in our assay is also conserved in other hemp and marijuana cultivars that were not tested by PCR here but for which the genome sequences are available. We aligned the X and Y chromosomal versions of *CsPDS5* regions from different cultivars: the marijuana cultivars ‘Ace High 3–2’ and ‘SourDiesel’, the high CBD hemp cultivars ‘Pink Pepper’ and ‘GoldenRedwood’ and a US hemp landrace variety named ‘BooneCounty’ (Lynch et al. 2025), and compared to the *CsPDS5* sequence of ‘Kompolti’, for which we successfully conducted the *CsPDS5-CAPS* assay (Fig. 2a). The alignment shows that the *CsPDS5* primer binding sites are conserved in all sequences, while the EcoRI restriction recognition site in *CsPDS5* is present on all analysed Y chromosomal but absent in X chromosomal sequences (Fig. 3). This demonstrates that the selected region is extremely conserved across the different cultivars and that the assay introduced here is potentially useful for a wide variety of *C. sativa* cultivars and landraces.

## Discussion

Here, we described the identification of a highly conserved sex marker gene, *CsPDS5*, in *C. sativa* and the development of an affordable, robust and reliable PCR-based method that is consistent among cultivars, crosses, tissues and developmental stages.

The genotyping method consists of amplifying and digesting a region of the *CsPDS5* gene, which has an enzyme restriction site on the Y version of this gene that is absent on the X version. The assay only requires the PCR reagents, a thermocycler and the EcoRI enzyme, which makes it an extremely affordable method, as only basic instruments and reagents, present in every molecular biology laboratory, are needed.

**Fig. 4:**
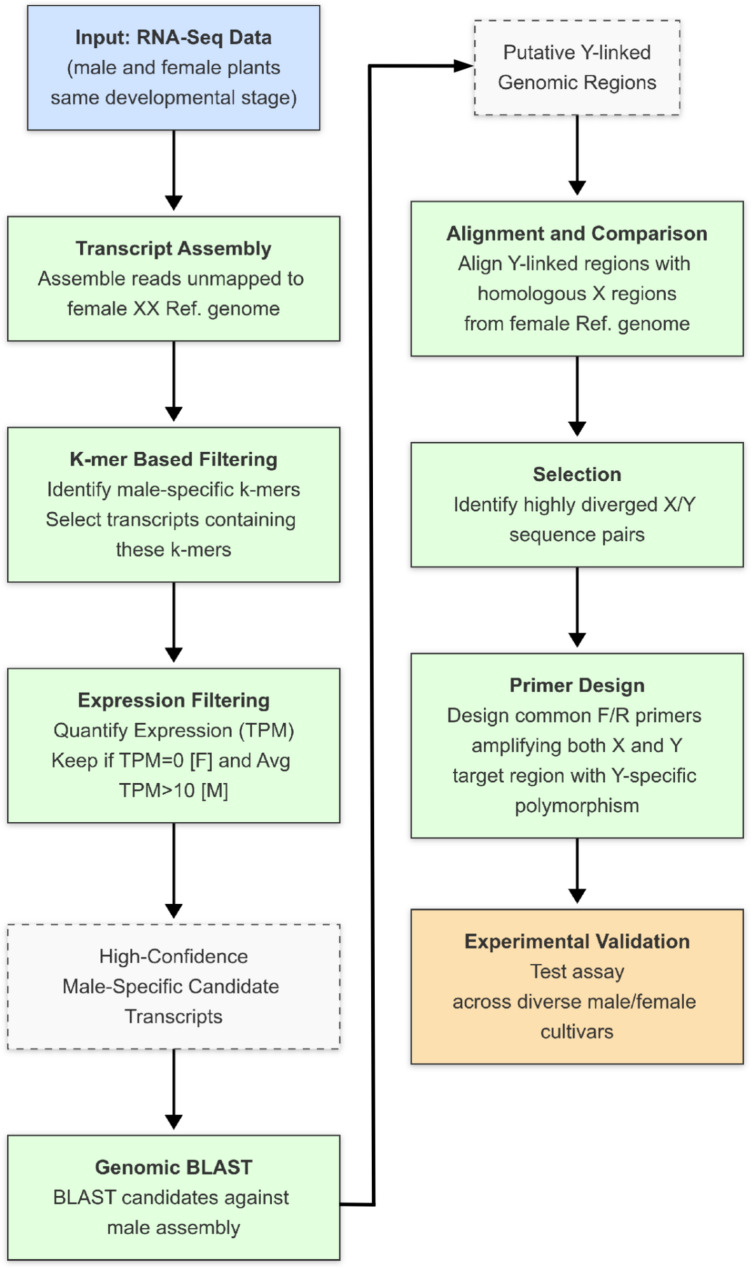
Pipeline used to identify molecular sex markers

The pipeline (Fig. 4) used here to identify the sex marker genes does not require an assembled Y chromosome, typically one of the more challenging tasks in genome assembly. Instead, gene expression data from male and female individuals are used to infer Y chromosomal transcripts, and genomic data are then leveraged to identify X and Y chromosomal gene copies. This basic workflow should apply to other species (plants and animals) with sex chromosomes and may therefore serve as a blueprint to identify molecular markers for sex in a range of different species. Although we leveraged the presence of a genome assembly of a male plant to retrieve the transcripts corresponding to the Y chromosome genomic sequence, the method can be applied also to species without genome assemblies if exploiting transcript polymorphisms instead of intron polymorphisms.

It is noteworthy that male-biased expression to identify Y chromosomal genes was an important step in our pipeline as no assembled Y chromosome was available, and it was hence difficult to identify genes encoded on the Y chromosome by other means. However, if a high-quality Y and X chromosome sequence is available, then identification of gametologs that have diverged in sequence might be a more viable approach.

We have tested the *CsPDS5-CAPS* assay on more than 500 samples from 14 hemp-type cultivars, six crosses, different tissues and developmental stages, and in all of the cases, the genetic and phenotypic sex matched, showing an accuracy of 100%. Analyses of publicly available genomes further demonstrated the presence of the EcoRI restriction site and primer binding sites also in other hemp and marijuana cultivars, indicating that the *CsPDS5* polymorphism is well conserved in *C. sativa*. Other *C. sativa* sex markers, like MADC6, are located in retrotransposons and may have experienced copy number variations and transposition (Toth et al. 2020). *PDS5* genes have an essential role in the function of the cohesin complex in many eukaryotes (Panizza, et al. 2000), and thus, *CsPDS5* might be a functional gene and not be subject to the same constraints as MADC6.

Also, since *CsPDS5* is present on both the X and the Y chromosomes, it simultaneously provides information about XX and XY genotypes using a single primer pair, which is different from other sex genotyping methods (Techen et al. 2010; Toth et al. 2020; Torres et al. 2022; Prentout, et al. 2025).

Our assay was developed to provide a robust sex genotyping method using basic laboratory equipment. However, we further adapted the assay into a high-throughput method using a TaqMan SNP genotyping assay, which was successfully validated on 22 ‘FINOLA’ samples, which could be used in larger breeding programmes. Given the assay is based on a 3 bp difference between X and Y chromosomal *CsPDS5* copies (Fig. 3), the marker is expected to be also suitable for other high-throughput methods such as PACE or amplicon sequencing.

The sex expression of *C. sativa* is known to be influenced by various factors, and the relative contribution of genetic vs. environmental factors for sex expression remains somewhat unclear (reviewed in Schilling et al. 2020). The observation that our molecular sex marker was in complete agreement with the phenotypic sex for hundreds of samples, including a mapping population that originated from a cross of a dioecious with a monoecious cultivar, illustrates that genetic components play a very strong role in sex determination in *C. sativa*. Unquestionably, external factors like the application of silver nitrate, an ethylene inhibitor that leads to the development of male flowers on female plants, can modify the sex expression (Galloch 1978; Truta, et al. 2007). However, our observations indicate that under conventional growth conditions, sex is almost solely determined by the XY sex determination system.

## Material and methods

### Gene selection

The pipeline to identify Y chromosomal transcripts is outlined in (Shi et al. 2025). This pipeline leverages RNA-seq data from male and female individuals, comparative k-mer analysis, and filtering criteria to isolate candidate Y-linked genes with higher confidence (Shi et al. 2025).

Briefly, the first step of the method is to assemble the transcripts from the reads that fail to map to the reference female XX chromosome genome, as at least part of those are likely to originate from the diverged genes on the Y chromosome. Then, to further refine the resulting transcripts from false positives, we employed a k-mer-based method selecting only the transcripts containing 16-mers present in every male sample, but absent in every female sample. Finally, the transcripts are increasingly refined by quantifying their expression level and selecting the ones showing a statistically significant male bias, with filtering parameters of log2 fold change (log2FC) ≥ 1 and false discovery rate (FDR) *P*-value ≤ 0.05.

To further reduce the possibility of false positives, for example, caused by SNPs that occurred by chance only in male individuals, resulting in male k-mers not truly from the Y chromosomes, here we selected as candidate genes for the genotyping assay only transcripts presenting a TPM level of 0 in females and a relatively significant expression in males (mean TPM > 10). This stringent filter criterion aligns with the expected expression pattern of Y chromosomal genes, which should be entirely absent from XX genomes and therefore generate no mapped reads in female samples.

After identifying male-specific sequences, the whole genomic sequence was inferred by BLASTing the mRNA sequence against the JL Lion father genome (Genome ID: GCA_013030025.1). We considered as candidates for the PCR assay the sequences with identity and coverage scores close to 100% in a single, unambiguous hit to ensure specificity to the Y chromosome. Sequences producing multiple alignments or identical hits in a female genome, presumed to originate from autosomal or X-linked regions, were not considered as candidates for the PCR essay. As a last step, the whole genomic region, comprising introns, from the reference genome chromosome X and JL Lion chromosome Y, was aligned, and the genes showing the highest divergence in sequence between their chromosome X and chromosome Y versions were selected as the most suitable candidates.

### DNA isolation

The DNA was isolated from the corresponding sample using the DNeasy® Plant Mini Kit (Qiagen GmbH, Germany) according to the manufacturer’s instructions. DNA quality was checked by electrophoresis on a 1% agarose gel at 120 V for 20 min, and DNA quantity was determined using a Nanodrop spectrophotometer (ND-1000).

### PCR primer design and conditions

Both versions of the *CsPDS5* gene, from the X and the Y chromosome, were aligned using Clustal Omega and this alignment was used to manually design the primer pair (Table 2), the specificity of which was checked against the NCBI database using the BLASTn tool. The primers amplify a fragment of 419 bp for both versions of the gene. The primers were purchased from IDT. The IDT primer stocks were then used to create stock solutions with concentrations of 50 µM.

Although there are two SNPs in the forward primer region and one in the reverse, these are consistent in all X and Y sequences across cultivars (Fig. 3), so were taken into account during primer design by incorporating degenerate (mixed) bases (Table 2).

**Table 2:** Name and nucleotide sequences of the genotyping primers

Primer name	Nucleotide sequence 5’—> 3’
PDS5_EcoRI_fwd	GGAYTATCATCCAGAGAMTG
PDS5_EcoRI_rv	TACCATATTCTCATCAGARGC

PCRs were conducted in a final volume of 25.5 μL with: 17.75 μL nuclease-free water, 5 μL 5X Phusion HF Buffer (Thermo Fisher Scientific) which provides 1.5 mM MgCl_2_ in the final 1X concentration, 0.5 μL of dNTPs (10 mM each) (VWR Chemicals), 0.5 μL PDS5_EcoRI_fwd (50 μM), 0.5 μL PDS5_EcoRI_rv (50 μM), 0.25 μL of Phusion DNA Polymerase (2 U/μL) (Thermo Fisher Scientific) and 1 μL of DNA template (≥ 2 ng/μL).

The Biometra T3000 Thermocycler was used for the PCR. After an initial denaturation at 98 °C for 30 s, 40 cycles at 98 °C for 10 s, at 56.5 °C annealing temperature for 30 s, and an extension at 72 °C for 30 s were performed before a final extension at 72 °C for 5 min.

### EcoRI digestion

After the PCR, the products were subjected to a digestion with the restriction endonuclease EcoRI which recognises the sequence 5’-GAATTC-3’. The digestion reaction consisted of 1.3 μL of 10X FastDigest Green Buffer (Thermo Fisher Scientific), 1 μL FastDigest EcoRI (Thermo Fisher Scientific), 12.5 μL of the unpurified PCR product and 4.5 μL of nuclease-free water. The mix was incubated at 37 °C for 15 min.

### Gel electrophoresis

The digestion products were loaded on a 2% agarose gel containing SYBR safe DNA gel stain (invitrogen) and run for 30 min at 100 V. The gel was then visualised under UV.

### TaqMan real-time PCR setup and primer/probe design

Using the alignment of the X and Y chromosome sequences of the *CsPDS5* gene, specific primers (ordered from IDT) and Custom TaqMan™ MGB probes (Thermo Fisher Scientific) were designed to differentiate between the XX and XY genotypes (Table 3). One probe was designed to hybridise specifically to the X-linked allele (Fem probe), and the other to the Y-linked allele (Male probe). Both probes were labelled with a 5’ fluorescent reporter dye (FAM for the Male, and VIC for the Fem probe) and a 3’ non-fluorescent quencher (NFQ). Each probe also incorporated a MGB (Minor Groove Binder) at the 3’ end, which increases melting temperature and enhances binding stability, providing a better mismatch discrimination.

**Table 3:** Name and nucleotide sequences of the TaqMan assay genotyping primers and probes

Probe/primer name	Nucleotide sequence 5’—> 3’
XY_taqman_Fw1	TATTYCAWTGGAGCTTCTCACC
XY_taqman_Rv1	AACCTTMGKAGCACAKRTTTC
Male probe	AGTTACAGTTTGGAATTCCTTT
Fem probe	TGTCCGAATTACAGATTACATTT

The TaqMan assay was conducted in a final volume of 10 μL with: 5 μL 2 × TaqMan Fast Advanced Master Mix (Thermo Fisher Scientific), 0.25 μL Male probe (10 μM), 0.25 μL Fem probe (10 μM), 0.2 μL of XY_taqman_Fw1 (45 μM), 0.2 μL of XY_taqman_Rv1 (45 μM) and up to 4.1 ul of genomic DNA (at a minimum concentration of 2 ng/μL in the final reaction mix). The remaining volume was adjusted with nuclease-free water to reach a total of 10 μL.

The QuantStudio 7 Flex Real-Time PCR System was used for the genotyping TaqMan assay. The thermal cycle protocol included an initial polymerase activation step at 95 °C for 10 min, followed by 40 cycles of denaturation at 95 °C for 15 s and annealing/extension at 55 °C for 1 min, concluding with a final post-read stage at 60 °C for 30 s.

## Supplementary Information

Below is the link to the electronic supplementary material.

Supplementary file1 (FA 41 KB) Data S1: 25 putatively Y chromosomal transcripts

Supplementary file2 (FASTA 10 KB) Data S2: Fasta alignment files of 6 candidate genes potentially suitable as sex markers in C. sativa.

Supplementary file3 (FASTA 76 KB)

Supplementary file4 (FASTA 3 KB)

Supplementary file5 (FASTA 19 KB)

Supplementary file6 (FASTA 21 KB)

Supplementary file7 (FASTA 92 KB)

## Data Availability

All relevant data can be found within the manuscript and its supporting material.
